# The Y271 and I274 Amino Acids in Reverse Transcriptase of Human Immunodeficiency Virus-1 Are Critical to Protein Stability

**DOI:** 10.1371/journal.pone.0006108

**Published:** 2009-07-03

**Authors:** Hao-Jie Zhang, Yong-Xiang Wang, Hao Wu, Dong-Yan Jin, Yu-Mei Wen, Bo-Jian Zheng

**Affiliations:** 1 Department of Microbiology, the University of Hong Kong, Hong Kong SAR, People's Republic of China; 2 Key Laboratory of Medical Molecular Virology, Institute of Medical Microbiology, Shanghai Medical College, Fudan University, Shanghai, China; 3 Department of Biochemistry, the University of Hong Kong, Hong Kong SAR, People's Republic of China; Institut Pasteur, France

## Abstract

Reverse transcriptase (RT) of human immunodeficiency virus (HIV)-1 plays a key role in initiating viral replication and is an important target for developing anti-HIV drugs. Our previous study showed that two mutations (Y271A and I274A) in the turn RT (Gln^269^-Arg^277^) abrogated viral replication, but the replication capacity and RT activity was discordant. In this study, we further investigated why alanine substitutions at these two sites would affect viral replication. We found that both RT activity and RT protein were almost undetectable in viral particles of these two mutants, although the Pr160^gag-pol^ mutants were properly expressed, transported and incorporated. Using protease inhibition assay, we demonstrated a correlation between the degradation of the RT mutants and the activity of viral protease. Our native gel analysis indicated that the mutations at 271 and 274 amino acids might cause conformational changes, leading to the formation of higher order oligomers instead of dimers, resulting in increased protein instability and susceptibility to viral protease. Thus, residues 271 and 274 are critical to RT stability and resistance to viral protease. The conservation of the two amino acid residues among different strains of HIV-1 lent further support to this conclusion. The knowledge gained here may prove useful in drug design.

## Introduction

Reverse transcriptase (RT) of human immunodeficiency virus (HIV)-1, encoded by *pol* gene, is a multifunctional enzyme that possesses RNA- and DNA-dependent polymerase activities as well as RNase H activity [1]. RT is indispensable for HIV-1 and it converts the single-stranded viral RNA into double-stranded DNA upon viral entry into host cells. Due to its important role in viral life cycle, RT is one attractive target for antiviral drug design [2].

The biologically active form of HIV-1 RT is a heterodimer consisting of two subunits, p66 (66 kDa) and p51 (51 kDa). The p51 subunit is derived from p66 by proteolytic cleavage of its C-terminal domain [3]. The polymerase domain of p66 and p51, resembling a right hand configuration, consists of four subdomains, which are known as fingers, palm, thumb and connection. The fingers, palm and thumb subdomains of p66 form a nucleic acid binding cleft and the connection subdomains of the two subunits form the floor of the nucleic acid binding site [4]–[7].

The thumb subdomain has four α helices. Two antiparallel α-helices of them, α-H (Asn^255^ to Ser^268^) and α-I (Gln^278^ to Thr^286^), are important for holding the primer/template in position during the translocation in polymerization. The primary sequence (Val^254^ to Ala^288^) in the vicinity of these two α helices has been found to share homology with several other nucleic acid polymerases and has been termed the “helix clamp” [5], [8]. Extensive studies have been carried out to shed light on the relationship between the “helix clamp” and function of RT. The effects of alanine-scanning mutations in α-H and α-I on polymerase activity, primer/template (P/T) binding, fidelity and enzyme kinetics have been determined. While mutations in α-I do not affect P/T binding or fidelity significantly, several α-H mutants exhibit lower binding affinity, processivity and frameshift fidelity [9]–[12]. Previous studies have demonstrated that mutations in these two helices can have significant effect on RNase H activity, minus-strand DNA transfer activity and removal of polypurine track primer [13], [14].

Although alanine substitutions at sites 269, 270, 271 and 277 have been investigated in two studies [9], [10], detailed studies on the functional structure of the “turn” (Gln^269^-Arg^277^) between α-H and α-I were limited. In our previous study on hepatitis B virus RT, conserved residues located at the “turn” of helix clamp motif were found important for pregenomic RNA encapsidation during the assembly of nucleocapsids [15], [16]. Since this homologous helix clamp motif is also present in HIV-1 RT, we hypothesized that residues in the turn may play important roles in viral life cycle. Our recent study showed that alanine substitutions at 271 and 274 of HIV-1 RT drastically affected viral replication, but discordance between viral replication and RT activity was observed [17]. In this study, we confirmed our previous observations and further investigated why the two mutations abrogated viral replication. Our study demonstrated that these two mutations lead to rapid degradation of RT in viral particles, indicating that the residues of 271 and 274 are critical for maintaining the stability of HIV RT.

## Methods

### Plasmid constructs and cell lines

The parental HIV-1 proviral plasmids, pLAI.2, pNL4-3-ΔE-EGFP and pHEF-VSV-G, were obtained through the NIH AIDS Research and Reference Reagent Program, Division of AIDS, NIAID, NIH [18]–[20]. The pNL4-3-ΔE-EGFP based mutants (Y271A, G273A, I274A, K275A, V276A, R277A) and pLAI2 based mutants (Y271A and I274A) were constructed by site-directed mutagenesis using QuikChange II XL Site-Directed Mutagenesis Kit (Stratagene, USA) according to manufacturer's instruction. The open reading frame of RT p66 subunit was amplified from wild type or mutant pNL4-3-ΔE-EGFP using a pair of primers and cloned into pET-28b vector (Novagen, Shanghai) to obtain expression plasmid pET-p66 with the 6× His tag at 3′ terminus. The paired primers used for site-directed mutagenesis and construction of plasmids are listed in Table 1. Cell lines 293FT, HeLa and U373-MAGI-CXCR4_CEM_ [21] were maintained in Dulbecco's modified Eagle's medium (DMEM) supplemented with 10% fetal bovine serum and antibiotics (Invitrogen, USA). MT2 cells [22], [23] were maintained in RPMI 1640 supplemented with 10% fetal bovine serum and antibiotics.

**Table 1:** Primers for site-directed mutagenesis and RT p66 cloning.

Name	Sequences
	**Primers for site-directed mutagenesis**
**Y271A_F**	5′-GAATTGGGCAAGTCAGATTGCAGCAGGGATTAAAGTAAGGC-3′
**Y271A_R**	5′-GCCTTACTTTAATCCCTGCTGCAATCTGACTTGCCCAATTC-3′
**G273A_F**	5′-GGGCAAGTCAGATTTATGCAGCAATTAAAGTAAGGCAATTATG-3′
**G273A_R**	5′-CATAATTGCCTTACTTTAATTGCTGCATAAATCTGACTTGCCC-3′
**I274A_F**	5′-GGGCAAGTCAGATTTATGCAGGGGCAAAAGTAAGGCAATTATG-3′
**I274A_R**	5′-CATAATTGCCTTACTTTTGCCCCTGCATAAATCTGACTTGCCC-3′
**K275A_F**	5′-GTCAGATTTATGCAGGGATTGCAGTAAGGCAATTATGTAAAC-3′
**K275A_R**	5′-GTTTACATAATTGCCTTACTGCAATCCCTGCATAAATCTG AC-3′
**V276A_F**	5′-GTCAGATTTATGCAGGGATTAAAGCAAGGCAATTATGTAAAC-3′
**V276A_R**	5′-GTTTACATAATTGCCTTGCT TTAATCCCTGCATAAATCTGAC-3′
**R277A_F**	5′-CAGGGATTAAAGTAGCGCAATTATGTAAACTTCTTAGGGG-3′
**R277A_R**	5′-CCCCTAAGAAGTTTACATAATTGCGCTACTTTAATCCCTG-3′
	**Primers for p66 cloning**
**NcoI**	5′-TAACCCCCATGGGCCCCATT AGTCCTATTGAGACTG-3′
**XhoI**	5′-TGAGCAGTCTCGAGTAGTACTTTCCTG ATTC-3′

Mutated residues and the enzyme cleavage sequence are underlined.

### Preparation of pseudoviruses and live viruses

To prepare pseudoviruses and live viruses, 293FT cells were co-transfected with 7.5 µg wild-type or mutant pNL4-3-ΔE-EGFP, and 2.5 µg pHEF-VSV-G, or 10 µg wild-type or mutant pLAI.2 using Lipofectamine 2000 (Invitrogen, USA) according to the manufacturer's instructions. Supernatants were collected 48 h after transfection and stored at −80°C after spinning down and filtering to remove cell debris. The cells were rinsed with ice-cold phosphate-buffered saline (PBS), scraped from each plate and lyzed in cell lysis buffer (Boehringer, Germany) and 1× protease inhibitor cocktail (Roche, USA) on ice for 30 min. Cell lysates were stored at −20°C after centrifugation at 13,000×g at 4°C for 15 min to remove cell debris.

### One cycle infection assay

One cycle infection assay was carried out using normalized pseudoviruses as described previously [20]. Briefly, Jurkat cells (0.5×10^6^) were infected with viral supernatants containing 250 ng p24, which was measured by Vironostika HIV-1 antigen MicroELISA kit (Biomerieux bv Boxtel, Netherlands). The virus and cells mixture was spun at 1,800 *g* at 30°C for 2 h. After the 2-h spin infection, Jurkat cells were washed with 2 ml culture medium twice, then cultured in 24-well plates at 37°C for 48 h. The cells were collected and washed twice with PBS. After the cells were fixed with 1% paraformaldehyde in PBS for 30 min on ice, the infected cells, as determined by the expression of GFP, were measured using a FACSCalibur instrument (Becton Dickinson, USA) and analyzed with Cell Quest software (Becton Dickinson, USA) as described previously [20].

### Infection assays with live viruses

Infection with live viruses was conducted in U373-MAGI-CXCR4_CEM_ and MT-2 cells. U373-MAGI-CXCR4_CEM_ cells were infected with normalized wild type or mutant live viruses in 24-well plate as described previously [21]. Briefly, triplicate wells (6×10^4^ cells/well) were infected with live viruses (60 pg p24/well) which were diluted in DMEM containing 20 µg/ml of DEAE-dextran (Amersham Biosciences). After cultured at 37°C in 5% CO_2_ incubator for 48 h, the cells were fixed in 1% formaldehyde-0.2% glutaraldehyde in PBS for 5 min. After washing twice with PBS, the cells were stained with 400 µg/ml of X-Gal (5-bromo- 4-chloro-3-indolyl-β-D- galactopyranoside), 4 mM MgCl_2_, 4 mM potassium ferrocyanide, and 4 mM potassium ferricyanide in PBS for 2 h at 37°C. The plate was washed twice with PBS and blue foci were observed under microscope. Infection with live viruses was also carried out in MT-2 cells as described previously [22], [23]. In brief, MT-2 cells (1×10^4^ cells/well) in 96-well plate were infected with live viruses (20 pg p24/well). After cultured for 6 days, the virus-specific cytopathic effect (CPE) was observed under microscope.

### RT activity assay

The RT activity in pseudoviruses was measured by a RT assay using colorimetric kit (Roche, USA). Briefly, viral supernatants containing 2 µg p24 were centrifuged at 4°C for 2 h at 40,000×g and the viral pellets were resuspended in 50 µl lysis buffer. Lyzed viral pellets were 10 fold serially diluted and the subsequent procedures were carried out according to the manufacturer's instructions. RT activities of mutants were calculated and compared to that of wild type pseudovirus.

### Immunofluorescence microscopy

Expression and subcellular localization of precursor Gal-Pol polyprotein were detected by immunofluorescence microscopy as described previously with some modifications [24]. Briefly, HeLa cells cultured on coverslip in 24-well culture plate were transfected with 600 ng pNL4-3-ΔE-EGFP and 200 ng pHEF-VSV-G. Two days post-transfection, the cells were fixed with 500 µl 4% paraformaldehyde (PFA) at room temperature for 15 min. After washing 3 times with PBS, 500 µl of 50 mM ammonium chloride was incubated with the cells for 10 min to neutralize residual PFA. The cells were washed 3 times with PBS and treated with 0.05% Triton X-100 for 3 min. After washing 3 times with PBS, 500 µl 10% normal rabbit serum (NRS) in PBS was added to block the slip overnight at 4°C. Primary antibody (mouse monoclonal anti-integrase, 1∶100, Santa Cruz) and secondary antibody (Texas Red dye-conjugated Rabbit anti-mouse IgG, 1∶100, Jackson ImmunoResearch) were incubated with samples in dark at room temperature. Following each incubation, samples were subjected to 3 washes with 1% NRS in PBS. With 3 additional PBS washes, the coverslip was mounted using fluorescence mounting medium (Dako) and observed under LSM510 Meta confocal microscope (Carl Zeiss).

### Western blotting

Gal-Pol polyprotein and its products were tested by Western blotting as described previously with some modifications [25]. Briefly, viral proteins were separated by 10% SDS-polyacrylamide gel electrophoresis (SDS-PAGE) and electro-blotted onto Hybond-P PVDF membrane (GE Healthcare, Bio-sciences). After blocking with 5% skim milk for 1 h at room temperature, the membrane was incubated with primary antibodies (Rabbit polyclonal anti-RT, 1∶3000; mouse monoclonal anti-RT, anti-intergrase, anti-protease or anti-capsid, 1∶500) for 1 h. Membrane was washed three times with PBS containing 0.1% Tween 20 (PBS-T) and then incubated with horseradish peroxidase (HRP)-conjugated secondary antibody (goat anti-rabbit IgG or goat anti-mouse IgG, 1∶4000) for 1 h. After the blots were rewashed three times in 0.1% PBST, signals were visualized using ECL Western blotting substrate reagents (Amersham Biosciences, USA) and KODAK BioMax Scientific Imaging Film (Eastman Kodak).

### Expression and analysis of RT p66

Wild type and mutant pET-p66 expression vectors were respectively transformed into *E. coli* BL21(DE3) and the expression was induced with 0.3 mM isopropyl β-D-1-thiogalactopyranoside (IPTG) when *E. coli* grew up to an OD600 of 0.7∼1.0. *E. coli* expressing p66 was spun down and re-suspended in the binding buffer (50 mM NaH_2_PO4, 300 mM NaCl, 10 mM imidazole). After the bacteria were disrupted by ultrasonication and centrifuged at 15 000 g for 30 min at 4°C, the wild type and mutant p66 in supernatants were purified using Ni-NTA magnetic agarose beads (Qiagen, Germany). The purified p66 proteins were subjected to NativePage Novex Bis-Tris gel analysis and Western blotting according to the protocol recommended by Invitrogen, USA.

### Sequence alignments and structural analysis

RT sequences of 1083 HIV-1 strains obtained from the HIV complete sequence database (http://www.hiv.lanl.gov/content/sequence/NEWALIGN/align.html) were aligned and compared. Based on the X-ray crystal structures of HIV-1 RT (1RTH) from the Research Collaboratory for Structural Bioinformatics Protein Data Bank (RCSB PDB), structural models of wild type and mutant RTs were analyzed using Swiss-PdbViewer software (http://spdbv.vital-it.ch/).

### Statistical analysis

Statistical analysis of RT activities was performed by Student's *t* test using Stata statistical software. Results were considered significant at *P*≤0.05.

## Results

### Y271A and I274A mutations abrogated viral replication

**Figure 1:**
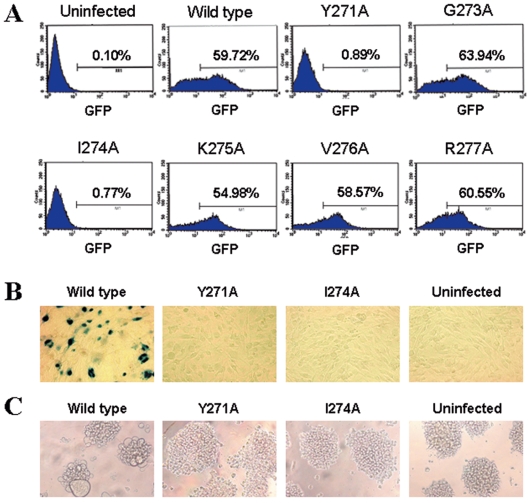
Infection with wild type and mutant pseudoviruses and live viruses. (A) Infection of Jurkat cells with wild type and six mutants of pseudovirus was detected by flow cytometry. The uninfected Jurkat cells (uninfected) were included as negative control. The infection was determined by the positive rate of infected cells expressing GFP, which was carried by the virus, at 48 h post-infection. Values presented in this figure are representative results of 3 independent experiments. (B) Infection of U373-MAGI-CXCR4_CEM_ cells with wild type as well as Y271A and I274A mutants of live HIV-1 virus. The infection was determined by the number of blue-staining foci (infectious units) in the cells at 48 h post-infection (original magnification of microscope: 200×). (C) Infection of MT2 cells with wild type as well as Y271A and I274A mutants of live HIV-1. The infection was assessed by observation of virus specific cytopathic effect (CPE) in the cells on day 6 post-infection. Cells were observed under fluorescent microscope (original magnification: 100×).

The effect of six RT mutations at the helix clamp turn in HIV-1 RT on viral replication was tested with pseudoviruses. When Jurkat cells were infected with the wild type and mutant pseudoviruses (250 ng p24 virus/5×10^5^ cells), viral replication was almost completely inhibited in the mutants Y271A and I274A, while replication of other mutants did not show significant difference as compared to that of the wild type (Fig. 1A). A similar result was also obtained when the cells were infected with lower amount (150 ng p24) of pseudoviruses (data not shown). To confirm the above results, replication of wild type, Y271A and I274A live viruses was further monitored in U373-MAGI-CXCR4_CEM_ and MT2 cells. Mutants Y271A and I274A were undetectable in U373-MAGI-CXCR4_CEM_ cells (Fig. 1B) and no virus-specific CPE was found in MT-2 cells (Fig. 1C). Since reverse transcription is the first step for HIV replication after viral entry into host cells, our results suggested that the two mutations might have affected the RT activity.

### RT activity and RT proteins were undetectable in pseudoviral particles of mutants Y271A and I274A

**Figure 2:**
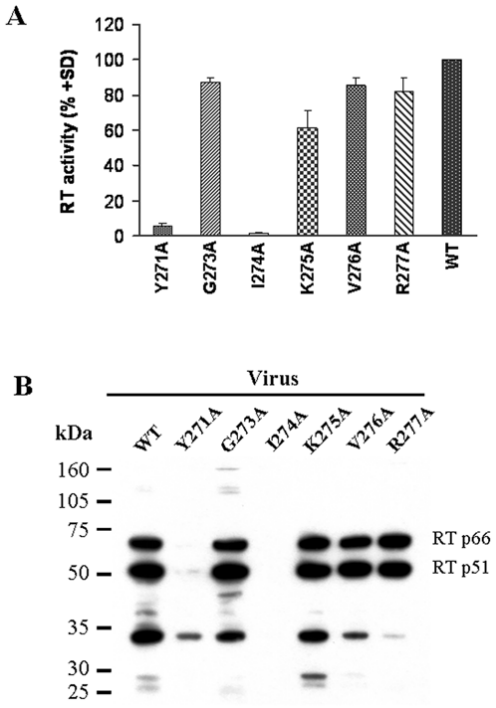
Detection of RT activity and RT protein in pseudoviruses. (A) RT activity in wild type (WT) and mutant pseudoviruses was tested by reverse transcriptase assay using a colorimetric kit. The viral particles in supernatant were concentrated and normalized (6 ng p24). The results are expressed as mean % of 3 independent experiments as compared to RT activity in wild type virus. (B) RT protein in lysed viral particles was detected by Western blotting using rabbit polyclonal anti-RT antibody. The assays showed that both RT activity and protein in mutant pseudoviruses Y271A and I274A were almost undetectable.

We thus interrogated the pseudovirus mutants for RT activity. The RT activity recovered from mutants Y271A and I274A were almost unnoticeable, as compared with that of wild type (Fig. 2A). This result was further confirmed using wild type, Y271A and I274A live viruses (data not shown). Next, we asked if the viral particles contain dysfunctional RT or the RT was not incorporated into the viral particles. By Western blotting, RT protein was basically undetectable in viral particles of these two mutants (Fig. 2B). The results suggested that the mutations could affect the incorporation of RT into the viral particles, leading to a defect in reverse transcription which initiates viral replication.

### Mutations Y271A and I274A did not affect Pr160^gag-pol^ expression and transportation

**Figure 3:**
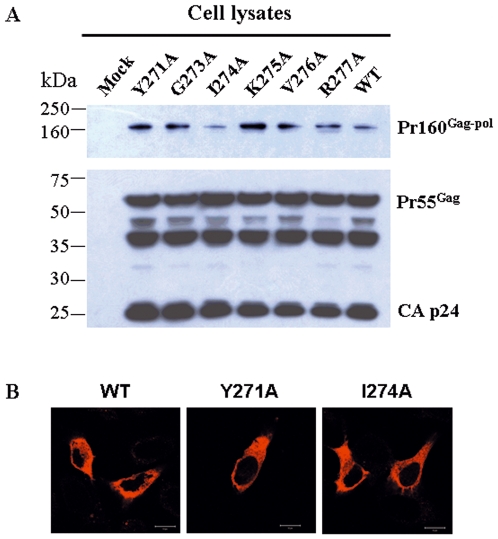
Expression and subcellular distribution of Gag-Pol polyprotein. (A) The expression of Gag-Pol polyprotein (Pr160^Gag-pol^), Gag protein (Pr55^Gag^) and capsid protein p24 (CA p24) in lysed cells transfected with pseudoviral vectors expressing wild type (WT) or six indicated mutants of RT was detected by Western blotting using mouse monoclonal anti-CA primary antibody and goat anti-mouse IgG HRP-conjugated secondary antibody. (B) The subcellular distribution of Pr160^Gag-pol^ in HeLa cells transfected with pseudoviral vectors for wild type (WT) RT or Y271A and I274A mutants were further detected by immunofluorescence staining using mouse monoclonal anti-integrase primary antibody and Texas Red dye-conjugated rabbit anti-mouse IgG secondary antibody. The positive cells were visualized under fluorescence microscope (original magnification: 400×, bar = 10 µm).

We next investigated whether the loss of RT in viral particles of the mutants was attributed to altered expression and transportation of the precursor protein, polyprotein Pr160^gag-pol^, during virus assembly. Pr160^gag-pol^ in lysates of cells transfected with the wild type and mutants was detected by Western blotting. As shown in Fig. 3A, similar levels of Pr160^gag-pol^, Gag protein (Pr55^Gag^) and capsid protein p24 (CA p24) were found in cells transfected with the wild type or mutant constructs, indicating that the expression and stability of RT precursor protein were not affected by the mutations. Immunofluorescence staining further demonstrated a normal subcellular localization of mutant Gag-Pol polyproteins as compared to that of the wild type, suggesting that the transportation of Gag-Pol was unlikely to be affected by alanine substitutions (Fig. 3B).

### The Gag-Pol polyprotein was incorporated into mutant virions of Y271A and I274A, but the RT was degraded by viral protease

**Figure 4:**
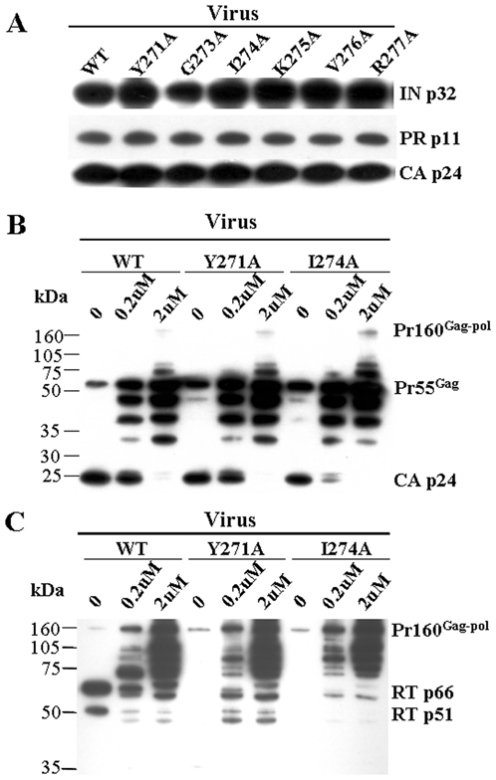
Detection of Gag-Pol polyprotein products in pseudoviral particles and the protease inhibition assay. (A) Cleavage products of Gag-Pol polyprotein, integrase (IN p32), protease (PR p11) and capsid protein p24 (CA p24) in wild type (WT) and mutant pseudoviruses were detected by Western blotting using mouse monoclonal anti-IN, anti-PR and anti-CA antibodies, respectively. (B) Pr160^Gag-pol^, Pr55^Gag^ and CA p24 were detected in wild type (WT) as well as Y271A and I274A mutant pseudoviruses produced in the absence (0) or presence of 0.2 and 2 µM protease inhibitor (indinavir) by Western blotting using mouse monoclonal anti-CA. (C) Pr160^Gag-pol^ and its subunits RT p66 and p51 were detected in wild type (WT) as well as Y271A and I274A mutant pseudoviruses produced in the absence (0) or presence of 0.2 and 2 µM protease inhibitor (indinavir) by Western blotting using mouse monoclonal anti-RT antibody.

To investigate whether the precursor Gag-Pol polyprotein was indeed incorporated into pseudoviral particles of mutants Y271A and I274A, products of the Gag-Pol polyprotein, integrase (IN), protease (PR) and p24, in wild type and mutant pseudoviral particles were examined by Western blotting. The results showed that, except for RT (Fig. 2B), all products of the Gag-Pol polyprotein, IN p32, PR p11 and CA p24, were detected in the mutants Y271A and I274A at a level similar to that of the wild type pseudoviral particles (Fig. 4A). Thus, Pr160^gag-pol^ was indeed incorporated into the virions and processed properly. To study if the RTs in the viral particles of Y271A and I274A mutants were degraded by proteolysis that made them undetectable, pseudoviruses of wild type and mutants were generated in the presence or absence of indinavir, a highly specific inhibitor of HIV-1 protease. Indinavir treatment was effective, because the treatment resulted in a dose-dependent inhibition of Pr55^Gag^ processing into p24 for wild type and mutant viruses (Fig. 4B). As shown in Fig. 4C, in the absence of indinavir, both RT p66 and p51 were readily detected in the wild type pseudovirus, but not in the mutant viruses. In the presence of indinavir, however, both RT p66 and p55 were markedly reduced in the wild type virus but became detectable in mutant Y271A viral particles, while RT p66 was also detectable in mutant I274A virus. We also detected the RT subunits in the viral supernatants collected at earlier time points (12, 24 and 36 hours post-transfection), but the mutant RTs were still undetectable (data not shown). These results suggested that the Y271A and I274A RTs were degraded after incorporation of Gag-Pol polyprotein into the virions, which might be attributed to the activity of viral protease.

### Y271A and I274A substitutions affected dimer-formation of RT

**Figure 5:**
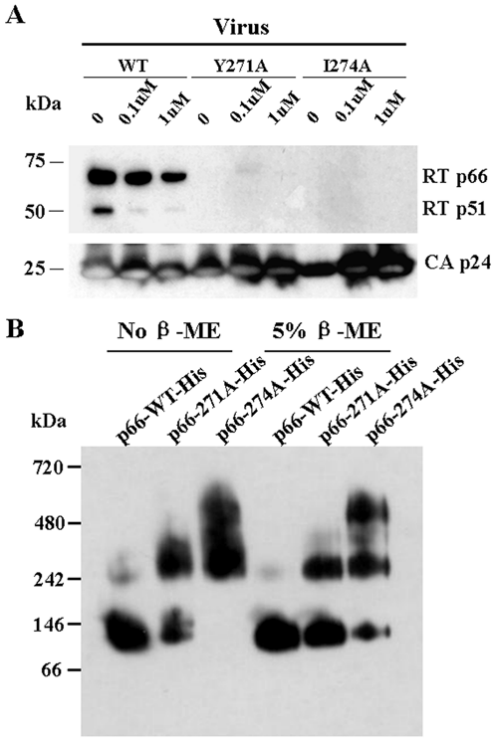
Detection of dimmer-formation of mutant RT. (A) RT was detected in wild type and mutant pseudoviruses generated in the presence and absence of EFV, the most potent dimerization enhancer, by Western blotting using mouse monoclonal anti-RT and anti-CA p24 antibodies, respectively. (B) Purified wild type and mutant RT subunit p66 were analyzed by native gel electrophoresis followed by Western blotting using mouse monoclonal anti-RT antibody in the presence or absence of β-mercaptoethanol (β-ME). Compared with wild type p66, which basically existed as homodimer, p66 subunit of the 271A and 274A mutants formed higher order oligomers, suggestive of conformational change.

Since RT dimer is the stable form which remains resistant to the proteolysis, we tested whether treatment of dimerization enhancer could reduce the mutant RT proteolysis. Wild type and mutant pseudoviruses were generated in the presence or absence of Efavirenz (EFV), the most potent dimerization enhancer [26]. The results showed that the RT mutants were still undetectable in the presence of EFV (Fig. 5A). The conformation of the wild type and mutant RT p66 was further analyzed by native gel electrophoresis followed by Western blotting (Fig. 5B). Under native conditions, wild type p66 subunit formed homodimer. However, the formation of homodimer in mutant Y271A was markedly reduced and a higher order oligomer appeared, which might be tetramer according to its molecular weight, while mutant I274A existed as at least two higher order oligomers, which were likely tetramer and octamer based on their molecular weights. However, the exact nature of the oligomers formed by mutant p66 subunits remained to be clarified. Furthermore, the treatment with β-mercaptoethanol could partially disrupt the higher order oligomers and improve dimer formation. This result implicated that dimer formation might be necessary for the stability of RT and its resistance to proteolysis after incorporation of Gag-Pol polyprotein into the viral particles.

### Amino acids at 271 and 274 are relatively conserved and big side chains may be important in maintaining RT stability and resistance to proteolysis

**Figure 6:**
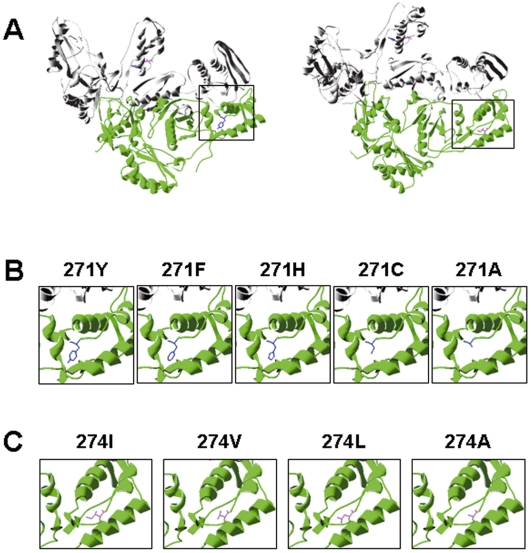
Structural models of naturally existent and artificially introduced substitutions of residues 271 and 274. We take p51 subunit as an example. (A) Locations of residues 271 and 274 in HIV-1 RT subunits p66 (grey) and p51 (green) (PDB coordinate 1RTH). The left panel shows a better view of residue 271 (blue); while the right panel reveals a better view of residue 274 (pink). (B) Structural models of naturally existent 271Y, 271F, 271H and 271C residues as well as the lethal 271A mutation of p51 subunit. The same area was highlighted above in the left panel of Fig. 6A. A big side chain (blue) is found in naturally occurring residues but not in 271A. (C) Structural models of naturally existing 274I, 274V and 274L residues as well as the lethal 274A mutation of p51 subunit. The same region was highlighted above in the right panel of Fig. 6A. A big side chain (pink) is found in naturally occurring residues I, V and L, but not in 274A.

By comparing 1083 complete sequences of HIV-1, it was found that the amino acids at 271 and 274 were relatively conserved. As shown in Table 2, tyrosine (Y) is the predominant naturally existent amino acid at 271 residue (99.19%), while phenylalanine (F), histidine (H) and cysteine (C) occur rarely (<1%). Similarly, isoleucine (I) is the predominant naturally existent amino acid at 274, which accounts for 98.43%, while in less than 2% of all cases, valine (V) and leucine (L) are found at this site. This finding suggested that these amino acids might probably play important role in maintaining the active conformation of RT. Consistently, structural analysis suggested that amino acids at these two sites are buried in the thumb region of RT (Fig. 6A). It was further revealed that all wild type RTs have relatively big side chains, but they are absent from the mutants 271A and 274A (Fig. 6B & 6C). Thus, the loss of the side chains in the RT mutants plausibly leads to conformational change of RT, leading to aberrance in dimer formation and susceptibility to proteolysis by HIV-1 protease.

**Table 2:** Frequencies of naturally existent amino acid residues at 271 and 274 of HIV-1 RT.

Location in HIV-1 RT	Amino acids	Frequency
	Y	99.19%
271	F	0.55%
	H	0.18%
	C	0.09%
	I	98.43%
274	V	1.48%
	L	0.09%

## Discussion

HIV RT plays a key role in viral replication and is an important target for development of anti-HIV drugs. However, the turn between helices H and I of HIV-1 RT thumb region (amino acid residues 269 to 277) has not been well characterized yet. To understand the structure-function relationship of this turn in details, we investigated whether alanine substitutions in this region would affect RT activity. Except for residues 269 and 270, which have been reported to have no significant influence on RT activity [9], [10], and for residue 272, which is originally an alanine, six mutant pseudoviruses (residues 271 and 273 to 277) were constructed and viral replication was compared with that of the wild type virus. The replication of two mutants, Y271A and I271A, were found to be almost completely abolished (Fig. 1A). This result was further confirmed in live virus system (Fig. 1B & 1C). Since it has been reported that bacterially expressed Y271A mutant has only 1% activity of wild type enzyme [9], we asked whether the mutants would similarly affect RT activity in the viral particles. The results showed that the RT activity was basically undetectable in viral particles of these two mutants (Fig. 2A). It was also found that the loss of RT activity in the viral particles might be attributed to the absence of RT in the viral particles rather than the incorporation of dysfunctional RT into the virions (Fig. 2B). After viral entry into the cells, the first step of HIV-1 replication is reverse transcription. Our results thus suggested that replication of mutant viruses was abrogated, because the mutant RT did not exist in the virion.

Since HIV-1 RT is incorporated into the virion in the form of Pr160^gag-pol^, which is transported to cell membrane where the virus is packaged, through interaction with Pr55^gag^ [27]–[30], we thus investigated whether the mutations would affect Pr160^gag-pol^ expression and transportation. Our results showed that Pr160^gag-pol^ was properly expressed and transported in cells transfected with mutant constructs, Y271A and I271A (Fig. 3), indicating that these mutations did not affect either the production of the precursor or the interaction between Pr160^gag-pol^ and Pr55^gag^. It was further found that the Pr160^gag-pol^ was indeed incorporated into the virions of these two mutants, because except for RT, all other products of Pr160^gag-pol^ including p24, protease and integrase [31], [32], could be detected in the viral particles of mutants Y271A and I271A (Fig. 4A). Although it has been reported that the domain between residues 183 and 305 of RT is likely responsible for RT incorporation [33], our study ruled out the involvement of the turn (Gln^269^-Arg^277^). Another report has suggested that two mutations in RT, L234D and W239A, led to premature cleavage of the Gag-Pol precursor and reduced levels of viral enzymes in the virions [34]. Our results also excluded that the mutations of Y271A and I271A affected the cleavage of the Gag-Pol precursor. By a viral protease inhibition assay using indinavir, we finally demonstrated that the RT mutants were degraded after incorporation of the precursor polyprotein into the virions and the degradation was associated with the activity of viral protease (Fig. 4C). Our results indicated that the mutations in residues 271 and 274 would reduce the stability of RT inside the viral particles, rendering it susceptible to viral protease.

Post-incorporation degradation of RT has been reported previously. Wapling et al. [35] have reported that mutations of W401L and W401A in RT can inhibit RT dimerization, resulting in reduced steady-state level of the RT subunits p66 and p51 in viral lysate, while Gag-Pol was not affected. They also showed that HIV-1 protease contributed to the degradation of RT subunits in the virions. A similar result was also observed when mutations were introduced into the protease cleavage site between RT p51 and RNase H domain [36]. Another mutation, L289K in p66 subunit, has also been reported to be able to abrogate dimerization [37]. Another group, when they tried to generate infectious molecular clones of Simian Immunodeficiency Virus, found that glutamic acid replacement at position 287 affected the stability of RT [38]. The biologically active and stable form of HIV-1 RT is a heterodimer consisting of p66 and p51, while the immediate precursor of p66/p51 heterodimer is the p66 homodimer. Proteolytic removal of the RNase H domain in one of the p66 homodimer subunit by HIV-1 protease leads to the formation of stable heterodimer p66/p51 [39]. RT exists as an equilibrium mixture of monomers and dimers that include the p66/p51 heterodimer and the p66/p66 and p51/p51 homodimers, among which the heterodimer is the most stable and the p51/p51 homodimer is the most unstable [40]–[43]. Plausibly, the degradation of RT as reported in previous studies may be ascribed to the inhibition of RT p66 dimerization by the mutations.

The mechanism of RT degradation in the virions observed in this study, however, may be different. Our results showed that RT mutants were still undetectable in the viruses generated in the presence of a potent dimerization enhancer (Fig. 5A). Our native gel analysis showed that the dimer form of RT mutant 271A was detected, although it was markedly reduced as compared to the wild type, while mutant 274A could not form dimer. Instead, higher order oligomers of RT were detected in both mutants (Fig. 5B). It has been reported that higher order oligomerization may occur in HIV-1 RT [41], [44], [45]. Our results have also indicated that Y271A and I274A substitutions may change the conformation of RT, leading to oligomerization. The higher order oligomers formed in these 2 mutants, perhaps tetramer and octamer, may be associated with instability of RT mutants and their susceptibility to viral protease. Furthermore, according to the locations of residues 271 and 274 (Fig. 6A), it is highly probable that alanine substitutions at 271 and/or 274 can affect the conformation of p51 thumb region and subsequently its interaction with RNase H domain of p66, resulting in the exposure of the 7-amino-acid p51-RNas H cleavage sequence in the p66 subunit [7], [46] and consequent proteolytic degradation by HIV-1 protease. The relative conservation of RT 271 and 274 residues and the loss of big side chains at these two sites in the mutants as shown in the structural models (Fig. 6B & 6C) also support their important roles. We tried to demonstrate this in vitro, by treating wild type and mutant RTs with recombinant protease, but wild type RT, as well as mutant RTs, could not be digested in vitro (data not shown). This result is consistent with that reported by Abram and Praniak [36]. This could be explained by different proteolytic stability of RT in vitro and in virions.

Taken together, our study showed that alanine substitutions at residue 271 or 274 of HIV-1 RT could cause conformational changes, rendering RT unstable and susceptible to viral protease. Thus, it is demonstrated that the “turn” (Gln^269^-Arg^277^) between two helices may be important in maintaining protein stability and formation of bioactive dimer. Mutations in residues 271 and 274 of RT may inactivate the virus, which may enter cells but can not replicate. These findings may prove useful in anti-HIV drug design and vaccine development. Considering the emergence of resistance to the current RT inhibitors, this turn, especially amino acid residues 271 and 274, may be an ideal new target for antiviral drug design. The agents targeting this region may be able to affect the stability or dimerization of HIV-1 RT and subsequently inhibit viral replication. Moreover, the potential drug resistant mutations in this region, especially at residues 271 and 274, may probably inactivate the virus, which prevents the appearance of drug-resistant virus.
